# Supplementary material for: Addressing challenges in speaker anonymization to maintain utility while ensuring privacy of pathological speech
Source: Commun Med (Lond). 2024 Sep 25;4:182. doi: 10.1038/s43856-024-00609-5 (PMC11424628; doi:10.1038/s43856-024-00609-5)
Supplement: Supplementary file 2 — Supplementary Information [file 43856_2024_609_MOESM2_ESM.pdf]

# Addressing challenges in speaker anonymization to maintain utility while ensuring privacy of pathological speech - Supplementary Information


Soroosh Tayebi Arasteh (1,2,3), Tomás Arias-Vergara (1), Paula Andrea Pérez-Toro (1), Tobias Weise (1,2), Kai Packhäuser (1), Maria Schuster (4), Elmar Noeth (1), Andreas Maier (1), Seung Hee Yang (2)

(1) Pattern Recognition Lab, Friedrich-Alexander-Universität Erlangen-Nürnberg, Erlangen, Germany.
(2) Speech & Language Processing Lab, Friedrich-Alexander-Universität Erlangen-Nürnberg, Erlangen, Germany.
(3) Department of Diagnostic and Interventional Radiology, University Hospital RWTH Aachen, Aachen, Germany.
(4) Department of Otorhinolaryngology, Head and Neck Surgery, Ludwig-Maximilians-Universität München, Munich, Germany.

**Correspondence**
Soroosh Tayebi Arasteh, PhD
Pattern Recognition Lab
Department of Computer Science
Friedrich-Alexander-Universität Erlangen-Nürnberg
Martensstr. 3
91058 Erlangen, Germany
Email: soroosh.arasteh@fau.de

# Supplementary Tables and Figures

Supplementary Table 1: Overview of some expected features for different clinical groups of the dataset.

| Features | Description | Dysarthria | Dysglossia | Dysphonia | Cleft Lip and Palate |
|---|---|---|---|---|---|
| Phonation | Control and use of vocal folds and glottal configurations. | Yes | Sometimes | Yes | Sometimes |
| Phonetic | Clarity and accuracy of speech sounds production. | Yes | Yes | No | Yes |
| Prosody | Patterns of stress and intonation in speech. | Yes | Sometimes | No | Sometimes |
| Hypernasality | Excessive nasal resonance due to velopharyngeal insufficiency. | Sometimes | Sometimes | No | Yes |
| Hyponasality | Reduced nasal resonance, typically from nasal obstructions. | Sometimes | No | No | Sometimes |
| Loudness | Volume level of speech. | Sometimes | Sometimes | Sometimes | Sometimes |
| Rate | Speed at which speech sounds are articulated. | Yes | Sometimes | No | Sometimes |

This table details the expected occurrence of different speech features across four clinical groups—Dysarthria[1-3], Dysglossia[4], Dysphonia[5], and Cleft Lip and Palate[6-9]. Each feature is evaluated for its presence in each disorder with labels Yes for consistently present, No for not associated, and Sometimes for variable presence. The label Sometimes specifically indicates that the manifestation of features like Loudness[2] can vary among individuals, reflecting the complexities of diagnosing and treating speech pathologies.

Supplementary Table 2: The effects of varying privacy levels on the utility of pathological speech.

| McAdams Coefficient | Dysarthria | | Dysglossia | | Dysphonia | | Cleft Lip and Palate | |
|---|---|---|---|---|---|---|---|---|
| | AUROC [%] | Accuracy [%] | AUROC [%] | Accuracy [%] | AUROC [%] | Accuracy [%] | AUROC [%] | Accuracy [%] |
| Original Data | 97.33±0.51 | 93.80±0.73 | 97.73±0.41 | 92.87±0.84 | 99.12±0.42 | 97.37±0.59 | 96.44±0.21 | 90.99±0.44 |
| 0.5 | 97.05±0.39 (p=0.0035) | 91.45±0.87 (p=$4.2 \times 10^{-19}$) | 98.62±0.25 (p=$1.8 \times 10^{-17}$) | 94.69±0.67 (p=$6.2 \times 10^{-16}$) | 96.60±0.64 (p=$1.1 \times 10^{-27}$) | 91.72±0.88 (p=$3.6 \times 10^{-37}$) | 96.04±0.29 (p=$1.8 \times 10^{-10}$) | 90.14±0.49 (p=$7.1 \times 10^{-11}$) |
| 0.6 | 96.05±0.65 (p=$1.9 \times 10^{-14}$) | 90.67±1.00 (p=$1.0 \times 10^{-22}$) | 97.69±0.44 (p=0.58) | 93.11±0.95 (p=0.19) | 98.02±0.51 (p=$1.4 \times 10^{-15}$) | 94.89±0.72 (p=$1.2 \times 10^{-23}$) | 94.07±0.29 (p=$1.4 \times 10^{-41}$) | 86.62±0.50 (p=$3.5 \times 10^{-41}$) |
| 0.7 | 94.06±0.92 (p=$1.6 \times 10^{-26}$) | 90.07±1.01 (p=$7.8 \times 10^{-26}$) | 98.68±0.23 (p=$1.0 \times 10^{-18}$) | 94.66±0.79 (p=$1.5 \times 10^{-14}$) | 97.62±0.47 (p=$1.1 \times 10^{-21}$) | 92.31±0.83 (p=$8.7 \times 10^{-36}$) | 97.56±0.20 (p=$1.3 \times 10^{-30}$) | 92.87±0.48 (p=$6.3 \times 10^{-25}$) |
| 0.8 | 96.29±0.60 (p=$2.4 \times 10^{-12}$) | 91.16±1.32 (p=$2.4 \times 10^{-16}$) | 97.55±0.28 (p=0.011) | 92.62±0.58 (p=0.086) | 97.71±0.57 (p=$1.4 \times 10^{-18}$) | 94.40±0.85 (p=$4.4 \times 10^{-25}$) | 98.62±0.12 (p=$5.3 \times 10^{-48}$) | 94.48±0.34 (p=$2.2 \times 10^{-40}$) |
| 0.9 | 98.27±0.28 (p=$3.3 \times 10^{-15}$) | 94.93±0.85 (p=$5.0 \times 10^{-19}$) | 98.12±0.31 (p=$3.6 \times 10^{-6}$) | 93.60±0.84 (p=$7.7 \times 10^{-5}$) | 98.63±0.35 (p=$8.2 \times 10^{-8}$) | 94.53±0.72 (p=$3.0 \times 10^{-26}$) | 97.48±0.19 (p=$1.1 \times 10^{-29}$) | 91.88±0.51 (p=$3.6 \times 10^{-12}$) |
| 1.0 | 98.48±0.39 (p=$6.3 \times 10^{-17}$) | 95.84±0.69 (p=$5.9 \times 10^{-19}$) | 98.52±0.24 (p=$1.6 \times 10^{-15}$) | 94.12±0.66 (p=$1.0 \times 10^{-10}$) | 96.19±0.59 (p=$1.0 \times 10^{-31}$) | 90.78±1.08 (p=$3.1 \times 10^{-37}$) | 97.15±0.17 (p=$3.2 \times 10^{-23}$) | 91.43±0.40 (p=$4.4 \times 10^{-6}$) |
| McAdams Coefficient | Sensitivity [%] | Specificity [%] | Sensitivity [%] | Specificity [%] | Sensitivity [%] | Specificity [%] | Sensitivity [%] | Specificity [%] |
| Original Data | 94.11±1.05 | 92.68±1.03 | 92.55±1.51 | 93.49±1.36 | 97.10±0.71 | 97.70±0.76 | 90.22±0.98 | 91.64±1.07 |
| 0.5 | 90.42±1.88 (p=$4.6 \times 10^{-16}$) | 91.60±1.55 (p=0.00017) | 94.15±1.13 (p=$3.2 \times 10^{-7}$) | 94.73±1.28 (p=$2.5 \times 10^{-5}$) | 92.46±1.54 (p=$4.1 \times 10^{-24}$) | 91.03±1.73 (p=$6.5 \times 10^{-29}$) | 90.32±0.94 (p=0.61) | 90.60±0.84 (p=$2.6 \times 10^{-6}$) |
| 0.6 | 89.25±1.84 (p=$5.9 \times 10^{-21}$) | 91.27±1.95 (p=$4.7 \times 10^{-5}$) | 91.67±1.82 (p=0.010) | 93.50±1.40 (p=0.97) | 94.67±1.08 (p=$1.8 \times 10^{-17}$) | 95.24±0.98 (p=$2.1 \times 10^{-18}$) | 85.40±1.58 (p=$4.3 \times 10^{-23}$) | 89.17±1.40 (p=$5.3 \times 10^{-13}$) |
| 0.7 | 86.40±1.85 (p=$1.7 \times 10^{-29}$) | 89.73±2.05 (p=$7.7 \times 10^{-12}$) | 94.61±1.49 (p=$1.5 \times 10^{-8}$) | 93.70±1.60 (p=0.48) | 92.24±1.37 (p=$8.7 \times 10^{-27}$) | 92.41±1.55 (p=$3.4 \times 10^{-26}$) | 92.50±0.74 (p=$2.3 \times 10^{-17}$) | 93.48±0.66 (p=$1.2 \times 10^{-13}$) |
| 0.8 | 90.41±1.80 (p=$1.3 \times 10^{-16}$) | 90.47±1.88 (p=$3.5 \times 10^{-9}$) | 93.39±1.30 (p=0.0045) | 92.13±1.55 (p=$2.7 \times 10^{-5}$) | 94.35±1.07 (p=$1.0 \times 10^{-19}$) | 94.61±1.04 (p=$9.3 \times 10^{-22}$) | 94.67±0.60 (p=$1.0 \times 10^{-30}$) | 94.65±0.65 (p=$8.7 \times 10^{-22}$) |
| 0.9 | 93.91±1.19 (p=0.39) | 94.71±1.27 (p=$1.9 \times 10^{-11}$) | 93.53±1.12 (p=0.00066) | 93.96±1.10 (p=0.065) | 93.94±1.42 (p=$1.6 \times 10^{-18}$) | 95.17±1.04 (p=$3.6 \times 10^{-18}$) | 89.35±1.25 (p=0.00040) | 93.85±0.99 (p=$3.1 \times 10^{-14}$) |
| 1.0 | 94.98±1.03 (p=0.00014) | 95.17±0.99 (p=$2.9 \times 10^{-16}$) | 94.51±1.17 (p=$3.9 \times 10^{-9}$) | 93.94±1.42 (p=0.11) | 90.90±1.28 (p=$1.6 \times 10^{-32}$) | 90.76±1.53 (p=$1.0 \times 10^{-31}$) | 92.57±0.87 (p=$9.7 \times 10^{-17}$) | 91.54±0.85 (p=0.63) |

This table presents the outcomes of applying randomized McAdams coefficient anonymization method to the pathological speech data. The McAdams coefficient was systematically adjusted from 0.5 to 1.0 (in increments of 0.1) and pretrained ResNet34[10] models were trained separately for classification of different disorders including Dysarthria, Dysglossia, Dysphonia, and Cleft Lip and Palate (CLP) and quantified using

AUROC, accuracy, sensitivity, and specificity. The results are presented as mean ± standard deviation. Statistical significance between original and anonymized data for these utility metrics was determined using a two-tailed unpaired t-test, with p-values noted. Training sets comprised n=168 speakers (Dysarthria detection), n=168 (Dysglossia detection), n=110 (Dysphonia detection), and n=887 (CLP detection). Corresponding test sets included n=73 (Dysarthria detection), n=73 (Dysglossia detection), n=49 (Dysphonia detection), and n=381 (CLP detection).

Supplementary Fig. 1: The proposed randomized pitch shift algorithm for anonymization.

```
for all speakers do
    extract log-Mel-spectrograms by performing data preprocessing as described in classification process;
    if speaker is a female then
        if age < 8 then
            S = a random number sampled from (-1.2, -1.0);
        else then
            S = a random number sampled from (-1.2, -0.8);
    else if speaker is a male then
        p = randomly sample a number from (0, 1);
        if p < 0.5 then
            S = a random number sampled from (-1.2, -0.8);
        else then
            if age < 10 then
                S = a random number sampled from (0.5, 0.8);
            else if 10 < age < 20 then
                S = a random number sampled from (0.6, 1.0);
            else then
                S = a random number sampled from (0.8, 1.2);
    shift the pitch with S steps (semitones);
    MEAN = a random number sampled from (0, 0.2);
    STD = a random number sampled from (0, 0.005);
    apply additive Gaussian noise with MEAN and STD;
    synthesize the resulting modified Mel-spectrogram using a pretrained Hi-Fi GAN model;
    if S > 0 then
        perform spectral gating noise reduction;
```

Supplementary Fig. 2: The utility and privacy results of the anonymization on the PC-GITA Spanish dataset[11].

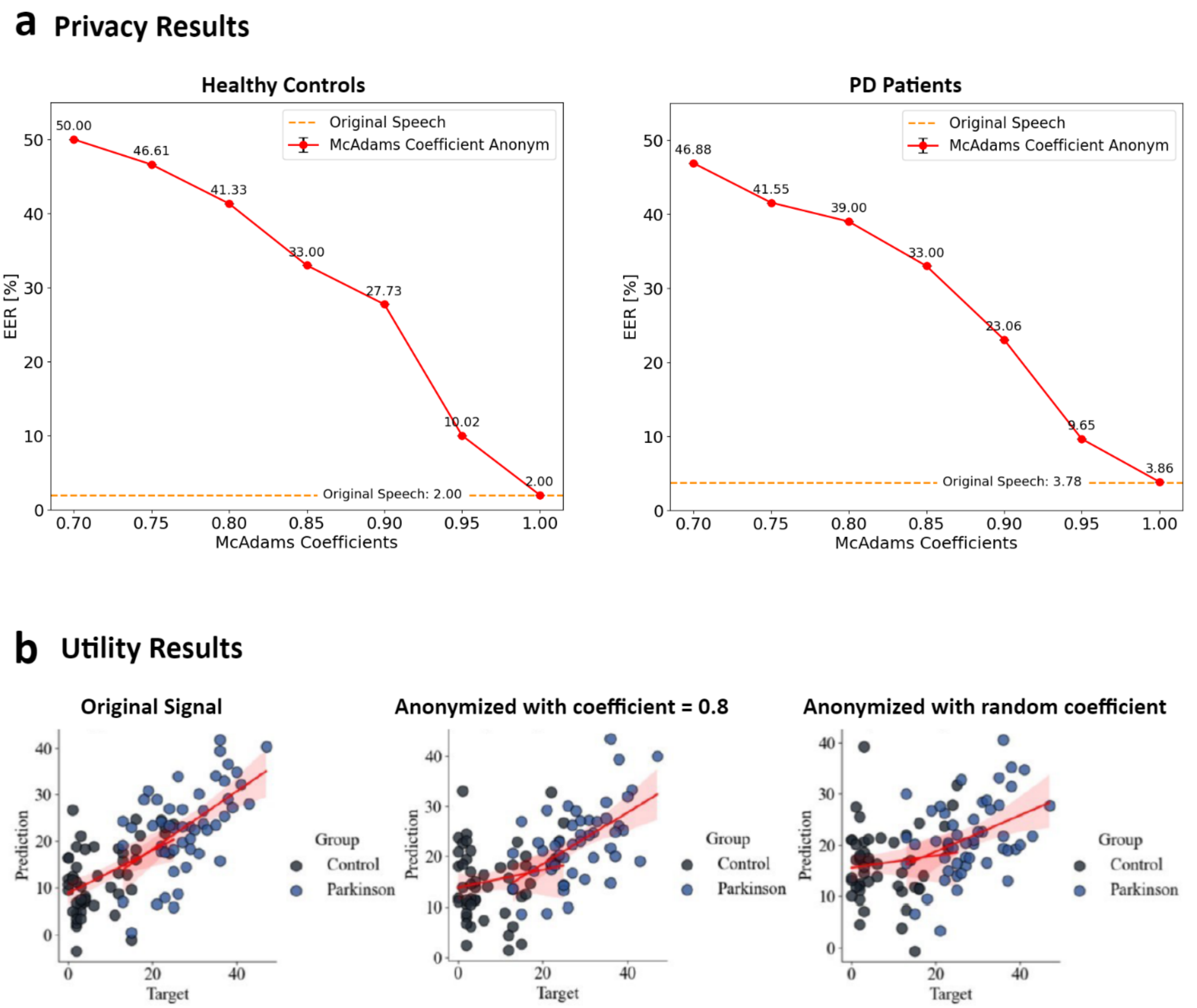


**a** The privacy results show the equal error rate (EER) values for both healthy controls and Parkinson's Diseases (PD) patients. Whiskers show the error bars, which indicate the standard deviation values. **b** shows the utility results where a linear support vector regression machine[12] was applied to predict the maximum phonation duration.

# Supplementary References

[1] Gupta, Siddhant, and Hemant A. Patil. "Analysis and Classification Dysarthric Speech." Biomedical Signal and Image Processing with Artificial Intelligence. Cham: Springer International Publishing, 2022. 167-182.
[2] K. Tjaden and G. E. Wilding. “Rate and loudness manipulations in dysarthria”. 2004.
[3] P. Enderby. “Disorders of communication: dysarthria”. Handbook of clinical neurology, Vol. 110, pp. 273–281, 2013.
[4] Schröter-Morasch, Heidrun, and Wolfram Ziegler. "Rehabilitation of impaired speech function (dysarthria, dysglossia)." GMS current topics in otorhinolaryngology, head and neck surgery 4 (2005).
[5] Roy, Nelson. "Functional dysphonia." Current Opinion in Otolaryngology & Head and Neck Surgery 11.3 (2003): 144-148.
[6] Wantia, Nina, and Gerhard Rettinger. "The current understanding of cleft lip malformations." Facial plastic surgery 18.03 (2002): 147-154.
[7] Millard, Tom, and Lynn C. Richman. "Different cleft conditions, facial appearance, and speech: relationship to psychological variables." The Cleft palate-craniofacial journal 38.1 (2001): 68-75.
[8] Rosanowski, Frank, and Ulrich Eysholdt. "Phoniatric aspects in cleft lip patients." Facial plastic surgery 18.03 (2002): 197-204.
[9] Harding, Anne, and Pamela Grunwell. "Characteristics of cleft palate speech." International Journal of Language & Communication Disorders 31.4 (1996): 331-357.
[10] He, Kaiming, et al. "Deep residual learning for image recognition." Proceedings of the IEEE conference on computer vision and pattern recognition. 2016.
[11] Orozco-Arroyave, J. R., Arias-Londoño, J. D., Vargas-Bonilla, J. F., González-Rátiva, M. C. & Noeth, E. New Spanish speech corpus database for the analysis of people suffering from Parkinson’s disease. in Proceedings of the Ninth International Conference on Language Resources and Evaluation (LREC’14) 342–347 (2014).
[12] Drucker, H., Burges, C. J. C., Kaufman, L., Smola, A. & Vapnik, V. Support Vector Regression Machines. in Advances in Neural Information Processing Systems 9 (NIPS 1996) (1996).
