# Supplementary material for: Addressing challenges in speaker anonymization to maintain utility while ensuring privacy of pathological speech
Source: Commun Med (Lond). 2024 Sep 25;4:182. doi: 10.1038/s43856-024-00609-5 (PMC11424628; doi:10.1038/s43856-024-00609-5)
Supplement: Supplementary file 3 — Description of Additional Supplementary Files [file 43856_2024_609_MOESM3_ESM.pdf]

Description of Additional Supplementary Files

File name: Supplementary Data 1
Description: The source data for Figures 3, 4, 5 and 6.
