# Supplementary material for: Addressing challenges in speaker anonymization to maintain utility while ensuring privacy of pathological speech
Source: Commun Med (Lond). 2024 Sep 25;4:182. doi: 10.1038/s43856-024-00609-5 (PMC11424628; doi:10.1038/s43856-024-00609-5)
Supplement: Supplementary file 5 — Reporting Summary [file 43856_2024_609_MOESM5_ESM.pdf]

Corresponding author(s): STA

Last updated by author(s): Sep 7, 2024

Reporting Summary

Nature Portfolio wishes to improve the reproducibility of the work that we publish. This form provides structure for consistency and transparency in reporting. For further information on Nature Portfolio policies, see our Editorial Policies and the Editorial Policy Checklist.

Statistics

For all statistical analyses, confirm that the following items are present in the figure legend, table legend, main text, or Methods section.

n/a	Confirmed	
☐	☒	The exact sample size (n) for each experimental group/condition, given as a discrete number and unit of measurement
☐	☒	A statement on whether measurements were taken from distinct samples or whether the same sample was measured repeatedly
☐	☒	The statistical test(s) used AND whether they are one- or two-sided<br>*Only common tests should be described solely by name; describe more complex techniques in the Methods section.*
☐	☒	A description of all covariates tested
☐	☒	A description of any assumptions or corrections, such as tests of normality and adjustment for multiple comparisons
☐	☒	A full description of the statistical parameters including central tendency (e.g. means) or other basic estimates (e.g. regression coefficient) AND variation (e.g. standard deviation) or associated estimates of uncertainty (e.g. confidence intervals)
☐	☒	For null hypothesis testing, the test statistic (e.g. F, t, r) with confidence intervals, effect sizes, degrees of freedom and P value noted<br>*Give P values as exact values whenever suitable.*
☒	☐	For Bayesian analysis, information on the choice of priors and Markov chain Monte Carlo settings
☐	☒	For hierarchical and complex designs, identification of the appropriate level for tests and full reporting of outcomes
☐	☒	Estimates of effect sizes (e.g. Cohen's d, Pearson's r), indicating how they were calculated

Our web collection on statistics for biologists contains articles on many of the points above.

Software and code

Policy information about availability of computer code

Data collection	To ensure transparency and facilitate further research, our entire source code is publicly accessible at https://doi.org/10.5281/zenodo.12806213. This repository includes comprehensive details on training protocols, evaluation procedures, data preprocessing, and anonymization processes, promoting reproducibility within the research community. The codebase is implemented in Python v3.9 and employs the PyTorch v1.13 framework for all deep learning tasks.
Data analysis	To ensure transparency and facilitate further research, our entire source code is publicly accessible at https://doi.org/10.5281/zenodo.12806213. This repository includes comprehensive details on training protocols, evaluation procedures, data preprocessing, and anonymization processes, promoting reproducibility within the research community. The codebase is implemented in Python v3.9 and employs the PyTorch v1.13 framework for all deep learning tasks.

For manuscripts utilizing custom algorithms or software that are central to the research but not yet described in published literature, software must be made available to editors and reviewers. We strongly encourage code deposition in a community repository (e.g. GitHub). See the Nature Portfolio guidelines for submitting code & software for further information.

Data

Policy information about availability of data

All manuscripts must include a data availability statement. This statement should provide the following information, where applicable:

- Accession codes, unique identifiers, or web links for publicly available datasets
- A description of any restrictions on data availability
- For clinical datasets or third party data, please ensure that the statement adheres to our policy

To ensure transparency and facilitate further research, our entire source code is publicly accessible at https://doi.org/10.5281/zenodo.12806213. This repository includes comprehensive details on training protocols, evaluation procedures, data preprocessing, and anonymization processes, promoting reproducibility within the research community. The codebase is implemented in Python v3.9 and employs the PyTorch v1.13 framework for all deep learning tasks.

Human research participants

Policy information about studies involving human research participants and Sex and Gender in Research.

Reporting on sex and gender

We evaluated the balance between privacy and fairness by analyzing demographic subgroups within our dataset. A fair classification network, in this context, is defined as one that maintains equal performance in detecting speech or voice disorders across all patient subgroups, both before and after anonymization. To assess this, we not only compared AUROC performance and EER privacy metrics across different subgroups but also employed statistical parity difference (PtD) as a measure of demographic fairness. This metric represents the accuracy disparity between minority and majority classes, with ideal values being zero—indicating no discrimination. Positive values suggest a benefit to the minority class, whereas negative values indicate potential bias against these groups. The demographic subgroups analyzed included gender (female and male) and age (adult and child), aiming to ensure equitable performance across these variables. Please refer to Table 1 for detailed characteristics of the dataset including sex and gender.

Population characteristics

The dataset used in our research comprised a wide array of speech utterances from across Germany. It featured a median participant age of 17, with a mean age of 30 years (± 25 years standard deviation), covering ages from 3 to 95 years. Table 1 offers an overview of the dataset demographics, including voice and speech disorder distributions, and gender breakdown.

Data collection
Data were collected from 2006 to 2019 during regular outpatient examinations at the University Hospital Erlangen and at over 20 different locations across Germany for the recording of control speakers. Every patient during a specialized consultation was invited to participate in the study. The study and the methods were performed in accordance with relevant guidelines and regulations and approved by the University Hospital Erlangen's institutional review board with application number 3473 and respected the Declaration of Helsinki. Informed consent was obtained from all adult participants as well as from parents or legal guardians of the children. Patients and control speakers were informed about the study's procedure and goals before consenting to participate and providing informed consent.
Recordings were made using a standardized procedure which included consistent settings, microphone setups, and speech tasks. Non-native speakers and patients whose speech was substantially disturbed by factors other than the targeted disorders were excluded. The Program for Evaluation and Analysis of all Kinds of Speech disorders (PEAKS), an open-source tool widely used in the German-speaking scientific community, was employed to document and manage the database. Recordings were captured at a 16 kHz sampling frequency and a 16-bit resolution, featuring subjects who are native German speakers, including various local dialects.

Speech features
The dataset included different causes with their main or prominent features of pathological speech, e.g., Dysphonia, refers to voice disorder containing phonation features, Dysglossia refers to articulation disorders containing mostly phonetic and sometimes phonation features, Dysarthria refers to speech disorder containing phonation, phonetic and prosody features, and CLP refers to speech and resonance disorder containing phonetic features, hyper- and hyponasality, and sometimes phonation features.

Recruitment

Data collection and prospective clinical studies were performed during routine outpatient visits at the University Hospital Erlangen. Informed consent was obtained from all adult participants as well as from parents or legal guardians of the children. Recordings were performed with a standardized procedure (setting, microphone, speech task) in each study.

Ethics oversight

The study and the methods were performed in accordance with relevant guidelines and regulations and approved by the University Hospital Erlangen's institutional review board with application number 3473 and respected the Declaration of Helsinki. Informed consent was obtained from all adult participants as well as from parents or legal guardians of the children. Patients and control speakers were informed about the study's procedure and goals before consenting to participate and providing informed consent.
The protocol for the PC-GITA dataset was approved by the Ethical Committee of the Research Institute in the Faculty of Medicine at the University of Antioquia in Medellín, Colombia (approval 19-63-673). All experiments were conducted in accordance with applicable national and international guidelines and regulations. Informed consent was obtained from all adult participants, as well as from the parents or legal guardians of the children involved. Our use of the PC-GITA dataset did not require separate ethical approval, as it is a restricted-access resource. Access was granted following our agreement to the dataset's user terms.

Note that full information on the approval of the study protocol must also be provided in the manuscript.

Field-specific reporting

Please select the one below that is the best fit for your research. If you are not sure, read the appropriate sections before making your selection.

☒ Life sciences ☐ Behavioural & social sciences ☐ Ecological, evolutionary & environmental sciences

For a reference copy of the document with all sections, see nature.com/documents/nr-reporting-summary-flat.pdf

Life sciences study design

All studies must disclose on these points even when the disclosure is negative.

Sample size	The dataset used in our research comprised a wide array of speech utterances from across Germany. It featured a median participant age of 17, with a mean age of 30 years (± 25 years standard deviation), covering ages from 3 to 95 years. Table 1 offers an overview of the dataset demographics, including voice and speech disorder distributions, and gender breakdown.
Data exclusions	To refine this dataset to a clean and unbiased selection, we adhered to all exclusion criteria mentioned in (https://www.nature.com/articles/s41598-023-47711-7), which encompassed data cleaning, ensuring speech quality and noise standards, and the elimination of multi-speaker utterances. Additional steps undertaken in this study include:<br>(1) Acknowledging the distinct speech characteristics between adults and children, we categorized the dataset into two primary subsets. Adults, defined as individuals over 20 years of age, were tasked with reading Der Nordwind und die Sonne, a phonetically rich German adaptation of Aesop's fable The North Wind and the Sun. This text comprises 108 words, 71 of which are unique. Conversely, children participated in the Psycholinguistische Analyse kindlicher Sprechstörungen (PLAKSS) test, which involved naming pictograms across slides, covering all German phonemes in various positions. Given the tendency of some children to describe pictograms with multiple words, and the occasional extra words between target words, recordings were automatically segmented at pauses exceeding 1s.<br>(2) Adults' subset focused on utterances characterized by Dysarthria, Dysglossia, and Dysphonia, alongside healthy control samples. Utterances with ambiguous or mixed pathologies or those representing conditions with a scant number of data points were excluded.<br>(3) For children, the emphasis was placed on utterances from individuals with CLP conditions — the most prevalent cranial malformation characterized by an incomplete closure of the vocal tract — as well as from healthy controls.
Replication	In a similar manner to our previous study using this dataset (https://www.nature.com/articles/s41598-023-47711-7), Considering each speaker contributed multiple utterances, and to account for the random sampling of utterances in training and testing, each test phase was repeated 50 times to reduce potential random biases, with evaluations strictly paired for a consistent comparison between anonymized and non-anonymized data.
Randomization	For each disorder subset and specific experiment, speakers were randomly allocated to training (70%) and test (30%) groups. This random allocation was consistent across experiments to ensure that the same training and test subsets were used for comparing anonymized data with original data, facilitating paired analyses to account for random variations. The division aimed to prevent overlap between training and test data. To address potential imbalances in the dataset, particularly where there was a limited number of healthy controls (81 in the adult subset), we adjusted the patient-to-control ratio. In cases of Dysarthria and Dysglossia with ample patient data (n=355 and n=542, respectively), we capped patient speakers at twice the number of controls. In the children's subset, which had more controls, we sampled controls up to 1.5 times the number of patients to maintain balance. The composition of the final training and test sets, ensuring a fair comparison between the two anonymization methods, was as follows: Training sets comprised n=168 speakers (Dysarthria detection), n=168 (Dysglossia detection), n=110 (Dysphonia detection), and n=887 (CLP detection). Corresponding test sets included n=73 (Dysarthria detection), n=73 (Dysglossia detection), n=49 (Dysphonia detection), and n=381 (CLP detection).
Blinding	Blinding was not relevant to our study given the nature and goals of the study.

Reporting for specific materials, systems and methods

We require information from authors about some types of materials, experimental systems and methods used in many studies. Here, indicate whether each material, system or method listed is relevant to your study. If you are not sure if a list item applies to your research, read the appropriate section before selecting a response.

Materials & experimental systems

n/a	Involved in the study	
☒	☐	Antibodies
☒	☐	Eukaryotic cell lines
☒	☐	Palaeontology and archaeology
☒	☐	Animals and other organisms
☐	☒	Clinical data
☒	☐	Dual use research of concern

Methods

n/a	Involved in the study	
☒	☐	ChIP-seq
☒	☐	Flow cytometry
☒	☐	MRI-based neuroimaging

Clinical data

Policy information about clinical studies

All manuscripts should comply with the ICMJE guidelines for publication of clinical research and a completed CONSORT checklist must be included with all submissions.

Clinical trial registration	The study and the methods were performed in accordance with relevant guidelines and regulations and approved by the University Hospital Erlangen's institutional review board with application number 3473 and respected the Declaration of Helsinki. Data collection and prospective clinical studies were performed during routine outpatient visits at the University Hospital Erlangen. Informed consent was obtained from all adult participants as well as from parents or legal guardians of the children.<br>The protocol for the PC-GITA dataset was approved by the Ethical Committee of the Research Institute in the Faculty of Medicine at the University of Antioquia in Medellín, Colombia (approval 19-63-673). All experiments were conducted in accordance with applicable national and international guidelines and regulations. Informed consent was obtained from all adult participants, as well as from the parents or legal guardians of the children involved. Our use of the PC-GITA dataset did not require separate ethical approval, as it is a restricted-access resource. Access was granted following our agreement to the dataset's user terms.
Study protocol	The study and the methods were performed in accordance with relevant guidelines and regulations and approved by the University Hospital Erlangen's institutional review board with application number 3473 and respected the Declaration of Helsinki. Data collection and prospective clinical studies were performed during routine outpatient visits at the University Hospital Erlangen. Informed consent was obtained from all adult participants as well as from parents or legal guardians of the children. For the speech tasks, patients who were non-native speakers were excluded. Recordings were performed with a standardized procedure (setting, microphone, speech task) in each study.<br>The protocol for the PC-GITA dataset was approved by the Ethical Committee of the Research Institute in the Faculty of Medicine at the University of Antioquia in Medellín, Colombia (approval 19-63-673). All experiments were conducted in accordance with applicable national and international guidelines and regulations. Informed consent was obtained from all adult participants, as well as from the parents or legal guardians of the children involved. Our use of the PC-GITA dataset did not require separate ethical approval, as it is a restricted-access resource. Access was granted following our agreement to the dataset's user terms.
Data collection	Data were collected from 2006 to 2019 during regular outpatient examinations at the University Hospital Erlangen and at over 20 different locations across Germany for the recording of control speakers. Every patient during a specialized consultation was invited to participate in the study. The study and the methods were performed in accordance with relevant guidelines and regulations and approved by the University Hospital Erlangen's institutional review board with application number 3473 and respected the Declaration of Helsinki. Informed consent was obtained from all adult participants as well as from parents or legal guardians of the children. Patients and control speakers were informed about the study's procedure and goals before consenting to participate and providing informed consent.<br>Recordings were made using a standardized procedure which included consistent settings, microphone setups, and speech tasks. Non-native speakers and patients whose speech was substantially disturbed by factors other than the targeted disorders were excluded. The Program for Evaluation and Analysis of all Kinds of Speech disorders (PEAKS), an open-source tool widely used in the German-speaking scientific community, was employed to document and manage the database. Recordings were captured at a 16 kHz sampling frequency and a 16-bit resolution, featuring subjects who are native German speakers, including various local dialects.
Outcomes	Not relevant to our study given the nature and goals of the study.
